# Identification of transcription factors dictating blood cell development using a bidirectional transcription network-based computational framework

**DOI:** 10.1038/s41598-022-21148-w

**Published:** 2022-11-04

**Authors:** B. M. H. Heuts, S. Arza-Apalategi, S. Frölich, S. M. Bergevoet, S. N. van den Oever, S. J. van Heeringen, B. A. van der Reijden, J. H. A. Martens

**Affiliations:** 1 Department of Molecular Biology, Faculty of Science, RIMLS, Radboud University, 6525 GA Nijmegen, The Netherlands; 2 Department of Laboratory Medicine, Laboratory of Hematology, Radboud Institute for Molecular Life Sciences (RIMLS), Radboud University Medical Center, 6525 GA Nijmegen, The Netherlands; 3 Department of Molecular Developmental Biology, Faculty of Science, RIMLS, Radboud University, 6525 GA Nijmegen, The Netherlands

**Keywords:** Molecular biology, Epigenetics, Transcription, Computational biology and bioinformatics, Gene regulatory networks

## Abstract

Advanced computational methods exploit gene expression and epigenetic datasets to predict gene regulatory networks controlled by transcription factors (TFs). These methods have identified cell fate determining TFs but require large amounts of reference data and experimental expertise. Here, we present an easy to use network-based computational framework that exploits enhancers defined by bidirectional transcription, using as sole input CAGE sequencing data to correctly predict TFs key to various human cell types. Next, we applied this Analysis Algorithm for Networks Specified by Enhancers based on CAGE (ANANSE-CAGE) to predict TFs driving red and white blood cell development, and THP-1 leukemia cell immortalization. Further, we predicted TFs that are differentially important to either cell line- or primary- associated MLL-AF9-driven gene programs, and in primary MLL-AF9 acute leukemia. Our approach identified experimentally validated as well as thus far unexplored TFs in these processes. ANANSE-CAGE will be useful to identify transcription factors that are key to any cell fate change using only CAGE-seq data as input.

## Introduction

DNA-binding proteins known as transcription factors (TFs) have crucial roles in gene expression regulation^1^. TFs can bind to specific cis-regulatory elements, such as enhancers and promoters, controlling transcriptomic machinery. Upon DNA binding, TFs can induce complex and dynamic cellular processes, ranging from cell fate determination, cellular reprogramming and cell cycle progression, to disease progression, and carcinogenesis^2^.

The powerful regulatory nature of TFs is exemplified by lineage-specific hematopoiesis; tight control of lineage-determining TFs will direct precursor cells to specific mature lineages. For example, GATA-1/2 or GFI-1B are essential for megakaryocyte/erythrocyte development, and SPI1 for myelopoiesis^3,4^. Also in leukemogenesis TFs have a major role. Acute myeloid leukemias (AMLs) are often plagued by recurrent mutations involving proteins with DNA binding properties^5^. Prime examples are EVI1 (MECOM), CEBPA, RUNX1, and HOXA9^6–8^.

Despite major advances in computational biology^9^, predicting mammalian TFs key to cell fate determination is still challenging. The cell’s constituents, such as DNA, RNA, and proteins, form complex regulatory networks and cannot be understood correctly if only discrete individual components are examined. To construct a reliable TF prediction model, the cell’s molecular state needs to be interrogated on a multitude of -omics disciplines. So far, different approaches have been developed to predict key TFs, some are either based on (differential) gene expression^10,11^ and/or co-expression information^12^. Other include state-of-the-art integrative strategies, such as integrating TF binding site information^13^. Unfortunately, systematic chromatin immunoprecipitation sequencing (ChIP-seq) for every TF in every cell type is a laborious and, at present, an unrealistic task. Predicting TF binding is therefore an excellent alternative to uncovering key TFs governing cell fate.

Cutting edge TF prediction incorporates (predicted) transcription factor binding sites and interactions between TF and their downstream target gene(s), also known as Gene Regulatory Networks (GRNs). Collectively, (predicted) TF binding site and GRN information can be used to predict key TFs in one cell state compared to another. This control in comparison design has been shown to be valuable in TF prediction^14^, even more so when incorporating TF binding profiles at enhancer regions^15^. For example, enhancer GRN-based method Analysis Algorithm for Networks Specified by Enhancers (ANANSE) is demonstrated to be a powerful tool for studying regulatory mechanisms. However, in order to use ANANSE to its full potential RNA-, ATAC-, and ChIP-seq (H3K27ac) data is required for at least two cell types.

Ideally, we want to reduce the necessity of multiple experimental setups while improving TF prediction by integrating information based on cis-regulatory elements, such as promoters, promoter-proximal regulatory elements, or enhancer elements, especially, when biological material is limited. One technique that uncovers a wide range of central gene regulatory elements is Cap Analysis Gene Expression sequencing (CAGE-seq)^16^. The method of capturing 5’-end steady-state capped RNAs identifies mRNA transcription start sites (TSSs), including non-characterized alternative TSSs, which are often tissue- and cell type-specific^17^; TSSs of long non-coding RNAs (lncRNAs); promoters accompanied by upstream antisense RNA (uaRNA); and enhancer RNAs (eRNAs). The latter two are characterized by bidirectional transcription^18,19^.

Here, we created an open source computational workflow that incorporates the enhancer network-based method ANANSE with the CAGE-seq analysis framework called CAGEfightR^15,16^ (ANANSE-CAGE). We demonstrated a strong correlation between bidirectional transcription and cis-regulatory elements, and its use in predicting cell type-specific TF binding profiles. Supervised modelling was used to improve TF binding prediction and cell conversion simulations were used to validate our findings. Next, we identified key TFs during induced erythroid differentiation, myeloid differentiation, and we predicted TFs important for immortalisation of MLL-AF9-driven leukemic cells. Subsequently, we predicted key TFs unique in either cell line- or primary- associated MLL-AF9-driven immortalisation, and key TFs in primary AML. The results demonstrated the wide use case of ANANSE-CAGE for studying development and pathology of haematological systems and its potential for uncovering novel key TFs and developing hypotheses.

## Results

### Bidirectional transcription initiation identifies active chromatin regions

**Figure 1:**
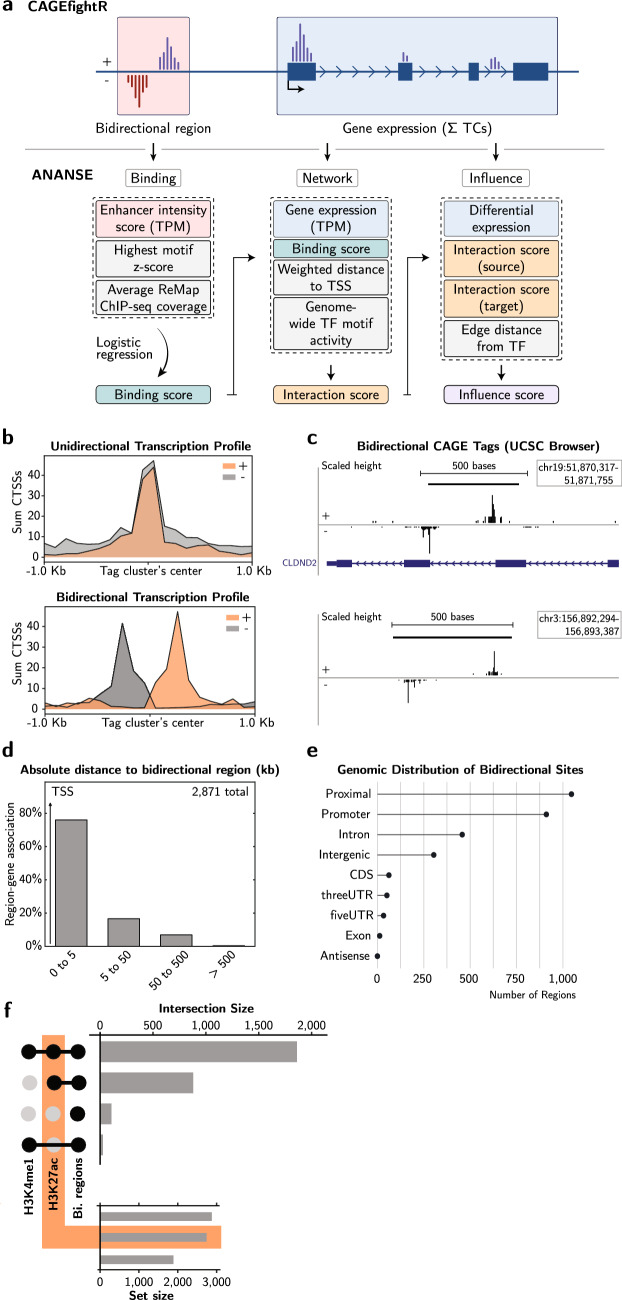
Bidirectional transcription initiation in cis-regulatory elements, including enhancers. (**a**) A schematic overview of the computational framework. First, bidirectional regions and gene expression are defined by CAGEfightR^16^. Gene expression is measured by summing all CAGE Tag Clusters (TCs) expressed per gene. Then, ANANSE is used to calculate a TF binding probability (binding score) by logistic regression using three types of data: enhancer intensity score in TPM (bidirectional regions), TF motif scores, and the average ReMap 2022 ChIP-seq coverage^15,21^, using Eq. (1). Gene expression (TPM), binding score, weighted distance to TSS, and genome-wide TF motif activity are then used to determine gene regulatory networks and its respective interaction score. The influence score is calculated based on differential expression (log2 fold change), edge distance from TF, and the interaction score of the source cell type and target cell type. The influence score represents how well the differences between two cell states can be explained by a TF, thus inferring importance for a cell’s state. The equations to calculate the scores, logistic regression, and GRNs were previously described by Xu et al.^15^. (**b**) Representation of summed CAGE-defined Transcription Start Sites (CTSS) expression at unidirectional (top) and bidirectional (bottom) transcription clusters. The orange color depicts the forward DNA strand ( + ) and in grey the reverse ( − ). (**c**) CAGE-seq tags representing bidirectional transcription in K562 cells for two separate genomic regions. (**d**) Bar graph representation of associated bidirectional regions with its closest gene. Y-axis shows the fraction of regions associated for its respective increment of kilobases (kb). (**e**) Number of bidirectional regions in K562 cells associated with various genomic locations. (**f**) Genome-wide co-occurrence (intersection size) of H3K4me1, H3K27ac, and bidirectional regions (Bi. regions) in K562 cells. The black dots represent intersection between the two ChIP-seq marks and bidirectional transcription. The set size represent the total amount of regions included.

5’-end capped RNA capturing methods, such as CAGE-seq, quantifies two types of transcription initiation events: one is defined as unidirectional transcription initiation, associated with Transcription Start Sites (TSSs), and the other is classified as bidirectional transcription initiation, which mark enhancer-associated regions (Fig. 1a).

To distinguish uni- and bidirectional transcription patterns, we quantified CAGE-defined TSSs (CTSSs) into Tag Clusters (TCs) using a slice-reduce approach in the myeloid leukemia cell line K562^16^. TCs manifesting gene expression were identified by an array of transcription events on the same strand, the expression of which was profiled to confirm this unidirectional transcription pattern (Fig. 1b, top). Similarly, distinct divergent transcription initiation patterns that were characterized by bidirectional transcription initiation at opposing strands were profiled (Fig. 1b, bottom and Fig. 1c). Using TCs expressed in at least 2 samples and removing weakly expressing TCs, our stringent criteria identified 2,876 high confidence bidirectional transcription regions.

Next, we investigated the various genomic loci that were accompanied by these bidirectional sites. In total 2,182 regions adjoined its nearest gene with an absolute distance between 0 and 5 kb (Fig. 1d). The remaining 689 regions were found in distal regions. We used UCSC’s Known Genes to annotate the relevant genomic regions (Fig. 1e). In total 1,957 bidirectional events were associated with proximal and promoter regions.

To confirm activity at bidirectional regions, we systematically calculated the number of intersecting regions between enhancer-specific histone modifications, H3 lysine 4 mono-methylation (H3K4me1) and histone H3 lysine 27 acetylation (H3K27ac), and bidirectional transcription regions. Nearly all bidirectional regions were either associated with both H3K4me1 and H3K27ac or H3K27ac alone (Fig. 1f), in line with enhancer studies using K562 cells^20^. In addition, we performed similar analyses for four other human cell lines: GM12878, HeLaS3, HepG2, and H1-hESC (Supplementary Fig. S1 and Supplementary Fig. S2a,b,c,d), corroborating these observations. Note that for all cell lines, the number of identified bidirectional regions is > tenfold lower than the regions marked by histone modifications (Supplementary Fig. S2e). Taken together, we identified a defined set of cis-regulatory elements associated with active chromatin modifications and bidirectional transcription in different cell systems.

### Curated transcription factor binding, motif scores, and bidirectional transcription activity accurately predict TF binding sites

**Figure 2:**
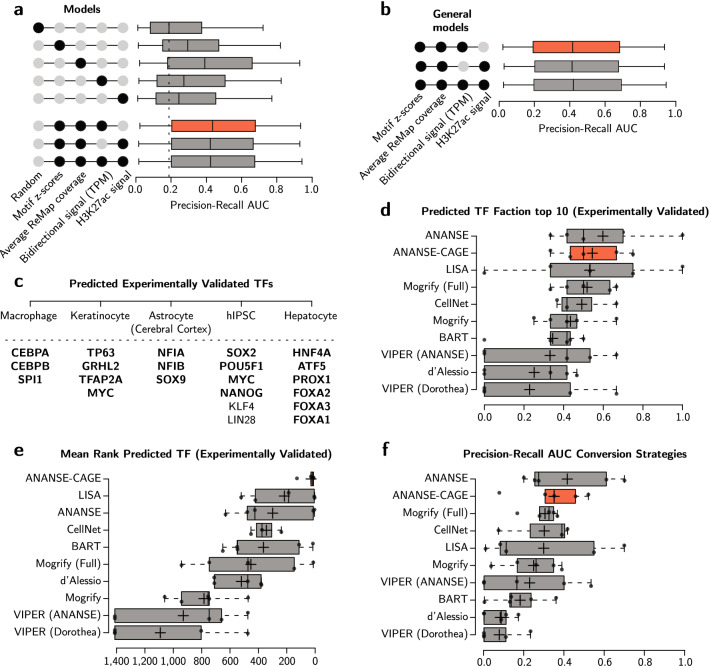
TF binding prediction performance and key TF prediction in cell conversion strategies. (**a**) TF binding prediction evaluation by Precision-Recall (PR) Area Under the Curve (AUC) in a boxplot. The performance of 230 TFs in five cell lines is cross-validated and plotted. Performance is compared to random sampling. All individual models perform significantly better than the random sampling method: TF motif z-scores (P Wilcoxon < 7.08e-07), average ReMap coverage (P Wilcoxon < 4.82e-18), bidirectional TPM (P Wilcoxon < 7.79e-05), and H3K27ac ChIP-seq (P Wilcoxon < 3.25e-03). Combined models are represented by the black dots. Similarly, all combined models perform significantly better than random sampling: TF motif scores, average ReMap coverage, and bidirectional TPM (P Wilcoxon < 3.08e-21); TF motif scores, average ReMap coverage, and H3K27ac ChIP-seq (P Wilcoxon < 2.30e-20); TF motif scores, average ReMap coverage, bidirectional TPM, and H3K27ac ChIP-seq (P Wilcoxon < 6.79e-21). The whiskers represent standard deviation, edges depict the inter-quartile ranges, and the black centre line illustrates the median. The model with the highest median score is depicted in red. (**b**) PR AUC for generalised models to predict TF binding for all other TFs in a boxplot. Performance is compared to random sampling as in (a). All general models perform significantly better than the random sampling method: TF motif scores, average ReMap coverage, and bidirectional TPM (P Wilcoxon < 1.02e-20); TF motif scores, average ReMap coverage, and H3K27ac ChIP-seq (P Wilcoxon < 7.98e-21); TF motif scores, average ReMap coverage, bidirectional TPM, and H3K27ac ChIP-seq (P Wilcoxon < 6.14e-21), visualized as in (a). (**c**) Summary of experimentally validated TFs in cell conversion strategies: skin fibroblasts to five different target cell types. TF predicted by ANANSE-CAGE are highlighted in bold. (**d**) Boxplot representing the fraction of (experimentally validated) TFs that are ranked in the top 10 per cell conversion strategy. Y-axis depicts the various TF prediction methods that are able to rank predicted TFs based upon their respective algorithm. Individual conversion types are depicted as dots. ANANSE-CAGE is depicted in red and other TF prediction methods are shown in grey. The whiskers represent standard deviation and edges depict the inter-quartile ranges. The plus sign represent the mean and the black centre line the median. (**e**) Boxplot representing the average inferred TF rank per cell conversion strategy for each of the TF prediction methods, visualized as in (d). (**f**) The PR AUC of the five cell conversions shown as a boxplot, visualized as in (d).

In order to investigate whether the bidirectional regions can function as a predictive measure for TF binding sites, we turned to logistic regression modelling. Specifically, a combination of four classifiers were modelled and tested: bidirectional transcription intensity (TPM); TF motif z-scores; average binding signal of TFs across cell types from the curated TF ChIP-seq database ReMap 2022^21^; and H3K27ac ChIP-seq signals in GM12878, HeLaS3, HepG2, H1-hESC, and K562 cells from ENCODE^22^. The predictive power of this set of models was determined (Fig. 2a) using the measure Precision Recall (PR) Area Under Curve (AUC), which is a performance measure when evaluating binary classifiers on imbalanced datasets. All models performed significantly higher compared to the random baseline, i.e. random guessing (median PR AUC 0.19, Wilcoxon Test). Out of the four classifiers, the average binding signal of TFs from ReMap 2022 contributed proportionally the most to the performance. The model including bidirectional TPM, motif scores, and average binding signal of TFs from ReMap showed the highest median (median PR AUC 0.44, P Wilcoxon < 3e-21), thus improved performance the most. Furthermore, a general model was trained which can be applied to all TFs that were not included in the supervised training (Fig. 2b). The general model also significantly improved performance (median PR AUC 0.42, P Wilcoxon < 1e-20). These results demonstrated that we significantly improved TF binding prediction at bidirectional regions using logistic regression modelling.

### Bidirectional transcription events predict key transcription factors for cellular reprogramming

To demonstrate the predictive capacity of ANANSE-CAGE, we assessed how our TF predictions compare to well-known experimentally validated TFs for a number of cellular reprogramming strategies. To this end, we established differential GRNs using CAGE-seq data from FANTOM5: fibroblasts (source cell type) and five different conversion cell types, including macrophages^23^, keratinocytes^24^, astrocyte derived from the cerebral cortex^25^, human induced pluripotent stem cells (hiPSCs)^26,27^, and hepatocytes^28,29^. In almost every case, we predicted the entire set of experimentally validated TFs (Fig. 2c, TFs in bold). For example, expression of SPI1, CEBPA, and CEBPB converted fibroblasts into macrophage-like cells^23^. The ANANSE-CAGE framework identified SPI1, CEBPA, and CEBPB within the top most important TFs for this conversion, corroborating these results. Likewise, established factors important in keratinocyte conversion (TP63, GRHL2, TFAP2A, and MYC) were amongst the top predicted TFs, confirming our predictions (Supplementary Table S1).

To further explore the performance of ANANSE-CAGE in its ability to rank predicted TFs, we compared our method with other TF prediction methods: ANANSE, Mogrify, LISA, BART, VIPER, CellNet, and the method of D’Alessio et al.^11,14,15,30–33^. Similarly, these methods rank predicted TFs, though with a different algorithm or type of data. We included three different performance metrics to confirm performance in five conversion strategies (macrophages, keratinocytes, astrocyte derived from the cerebral cortex, hiPSCs, and hepatocytes). First, by determining the fraction of (validated) TFs ranked in the top 10 highest scoring TFs per cell conversion strategy, we demonstrated the methods ability to rank (confirmed) important TFs. ANANSE-CAGE outcompeted nearly all other reported methods in this regard (Fig. 2d). Second, by examining the mean rank of all validated TFs per conversion, we illustrated the overall ranking performance. Similarly, ANANSE-CAGE exceled in ranking these validated TFs (Fig. 2e), with the majority ranking in the top 30 and nearly all in the top 50 (Supplementary Table S1). Third, when evaluating the PR AUC (Fig. 2f), a measure for evaluating prediction performance, ANANSE-CAGE was again amongst the highest performing methods.

Taken together, we showed that CAGE-defined bidirectional transcription regions can serve in prioritizing biologically relevant TFs using the ANANSE algorithm. Information about bidirectional transcription can significantly improve TF binding prediction as well as establish a prediction method that seems to outperform various other prediction methods.

### Identifying essential transcription factors during early, late, and definitive erythropoiesis

**Figure 3:**
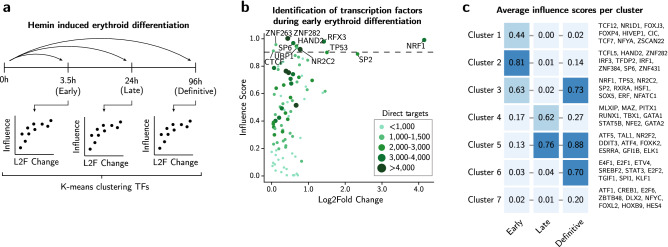
Temporal TFs during normal erythroid differentiation. (**a**) A schematic overview of k-means clustering of the inferred influence scores for three timepoints: early (3.5 h), late (24 h), and definitive (96 h). The top arrows represent the three ANANSE-CAGE analyses necessary for the unsupervised clustering. The scatterplots are a schematic representation of the scatterplot generated by ANANSE-CAGE depicting influence score, an inferred score for how well differences in two cell states can be explained by a TF, and log2 fold change. (**b**) Scatterplot representing inferred influence scores and log2 fold change. The color and size of the individual dots represent an approximation for the number of target genes that are calculated from the number of edges in differential GRNs. The dotted line represents a visual cut-off for highlighting the top ranked TFs. (**c**) Heatmap showing seven clusters of TFs during erythroid differentiation. The value in each square represents the average influence score of each cluster at each timepoint. To the right nine TFs from each cluster is depicted. More TFs are summarized in Supplementary Table S2. The color intensity represents the average influence scores.

To identify key TFs that drive erythroid differentiation, we applied ANANSE-CAGE to three stages of K562 hemin induced differentiation program: early (3.5 h), late (24 h) and definitive (96 h) (Fig. 3a and Supplementary Table S1). For each assay the influence scores are determined, a measure for importance of how a TF can explain transcriptional differences between two cell states. As an example, we depicted high influential TFs during early erythroid differentiation (Fig. 3b). Gene expression differences, amongst other factors (see Fig. 1a), contribute to calculating the influence score of predicted TFs. However, log2fold changes alone do not infer importance, see for example ZNF263 (Fig. 3b and Supplementary Table S1). In fact, this is a power of this prediction method. Likely, (high) gene expression changes are not always necessary for a TF to exert its function, as long as the respective TF is able to bind to the DNA. Next, we performed k-means clustering on the inferred scores of the predicted TFs (Supplementary Fig. 2f.). This revealed seven clusters of TFs that show similar temporal importance across the erythroid differentiation (Fig. 3c and Supplementary Table S2). Well documented TFs important in erythropoiesis, such as GATA1, GATA2, TAL1, KLF1, and GFI1B confirm our results^34–36^. TFs that have a thus far unknown role during erythroid differentiation also clustered according to temporal importance, e.g. HIVEP1 (early), MLXIP (late), FOXK2 (late-definitive), and RFX7 (definitive). Interestingly, GWAS studies suggest HIVEP1, MLXIP, FOXK2, and RFX7 to affect red blood cell phenotypes^37,38^. Together, we observed predictions that not only correlate well with known literature, it also provides a rich and instructive set of factors potentially essential for erythropoiesis that can be further explored.

### Discovering immortalisation-associated transcription factors in a MLL-AF9-driven leukemic cell line

**Figure 4:**
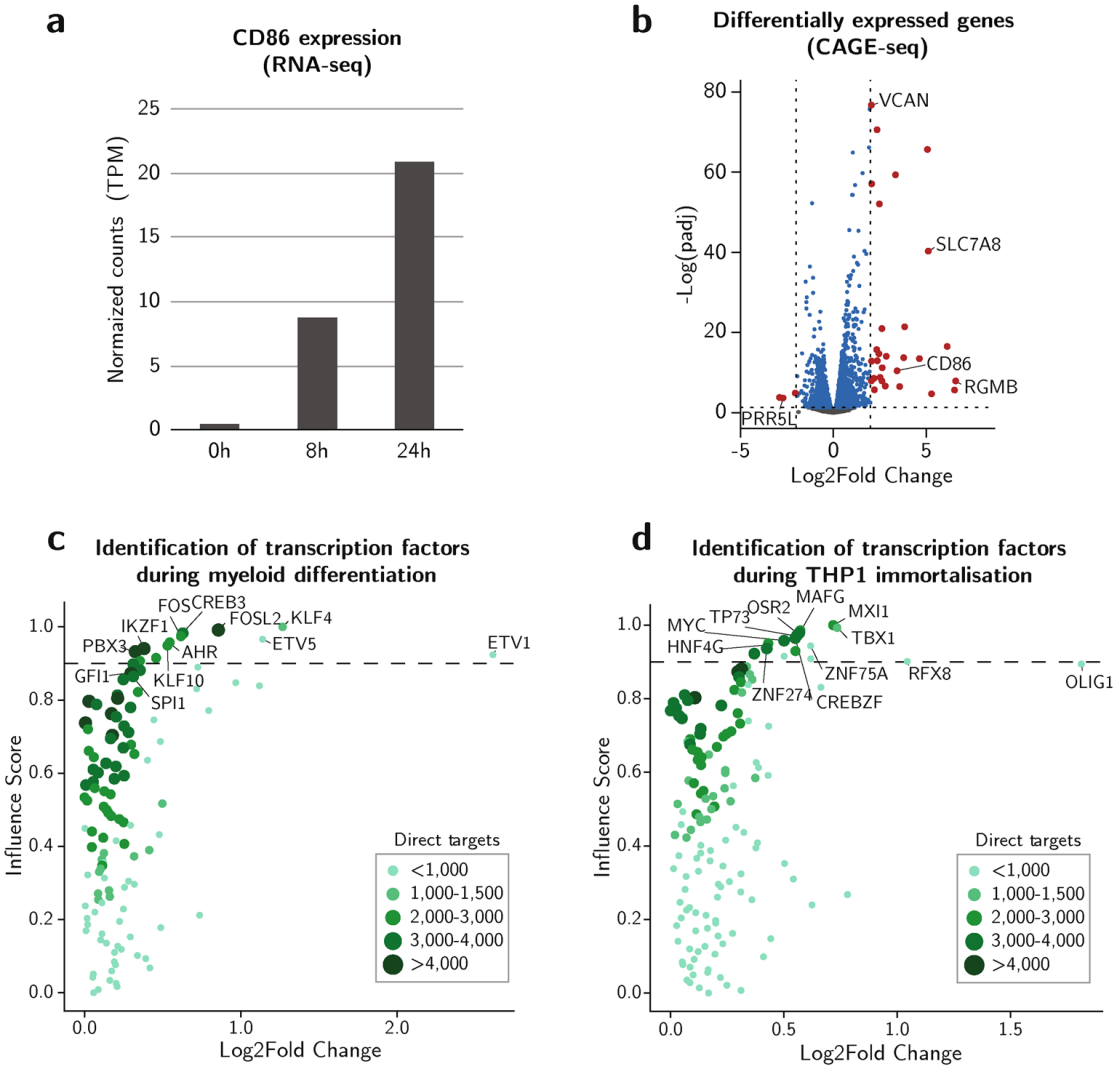
Key TFs in GSK-LSD1 induced early myeloid differentiation and immortalisation. (**a**) Bar graph depicting normalized (RPKM) CD86 expression (RNA-seq) in THP-1 after GSK-LSD1 induction (0 h, 8 h, and 24 h). (**b**) Differential expression of GSK-LSD1 induced THP-1 between 0 and 8 h (CAGE-seq). Y-axis depicts -log of the padj values and x-axis the log2 fold change. Dotted lines signify a cut-off for significance (padj < 0.05) and an absolute log2 fold change > 2. Genes significantly differentially expressed with a log2 fold change higher than 2 are depicted in red. (**c**) Scatterplot representing log2 fold change and inferred influence scores, an inferred score for how well differences in two cell states can be explained by a TF, in early myeloid differentiation. The color and size of the individual dots represent an approximation for the number of target genes that are calculated from the number of edges in differential GRNs. The dotted line represents a visual cut-off for highlighting the top ranked TFs. (**d**) Identification of TFs in THP-1 immortalisation, visualized similarly to (f).

One advantage of ANANSE-CAGE is that it can predict and rank important TFs in one cell state compared to another, e.g. comparing untreated to treated conditions, or vice versa. Here, we set out to predict important TFs in immortalisation of MLL-AF9-driven leukemic cells (untreated condition) and in early myeloid differentiation (treated condition). First, TFs that drive normal myeloid differentiation were determined. For this, we used a GSK-LSD1 inhibitor to induce myeloid differentiation in MLL-AF9 positive THP-1 cells^39^. Lysine-specific demethylase 1 (LSD1) is capable of interacting with MLL-AF9, sustaining leukemogenic potential of MLL-AF9 leukemic cells. Pharmaceutical inhibition of LSD1 results in induction of differentiation in these leukemic cells. This inhibition leads to monocyte differentiation accompanied with upregulated CD86 expression, a myeloid differentiation marker^40^. To validate the LSD1 inhibition, we first quantified CD86 expression^40^. Using RNA-seq and flow cytometry we observed a steady increase in CD86 expression over the course of 24 h (Fig. 4a and Supplementary Fig. S3a-b), in line with previous reports^41^.

Next, to determine early gene programs induced by LSD1 inhibition, we performed CAGE-seq analysis before and after 8 h GSK-LSD1 treatment and analysed the differentially expressed genes (Fig. 4b). This revealed 29 genes to be upregulated (*P* < 0.05, log2 fold change > 2) after GSK-LSD1 treatment and only 3 downregulated genes (*P* < 0.05, log2 fold change <  − 2). Despite the few differentially expressed genes (log2 fold change > 2) we were able to confirm significant higher CD86 expression upon GSK-LSD1 treatment, even at this very early timepoint. Importantly, CAGE-seq allowed the identification of bidirectional transcription sites (Supplementary Fig. S3c,d,e) and its corresponding expression values in addition to the gene expression information to generate a ranked list of putative driving key TFs.

We examined whether factors related to induced myeloid differentiation were predicted using ANANSE-CAGE. Among the top ranked TFs we find KLF4, GFI1, and SPI1 (Fig. 4c and Supplementary Table S1). KLF4, SPI1, and GFI1 are known critical regulators of monocyte differentiation^39,42^. Generally, ETS family TFs are recognized as key factors in governing haematopoiesis^43^. Here, we observed ETV1 to be amongst the highest scoring factors as well as showing high differential expression, leading to the hypothesis that ETV1 is important in GSK-lSD1 induced myelopoiesis.

In contrast to factors key in induced myeloid differentiation, we also predicted a set of TFs important in maintaining THP-1 cell immortalisation by determining differential GRNs between induced THP-1 (source type) and wild-type THP-1 (target type) (Fig. 4d and supplementary Table S1). Between the key TFs we found MYC , a well-known proto-oncogene involved in cell cycle regulatory mechanisms^44^, while other identified factors, such as OLIG1 and RFX8, do not have a reported role in MLL-AF9 driven leukemic cells. Though the high scores predict a potential role in THP-1’s oncogenic program for these novel factors. In summary, we find key TFs in MLL-AF9 driven immortalisation that can be further explored in more dedicated assays.

### Transcription factor prediction in MLL-AF9 positive primary AMLs

**Figure 5:**
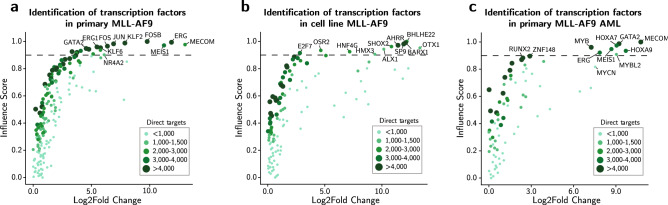
Identification of TFs in MLL-AF9-driven leukemia. (**a**) Scatterplot depicting log2 fold change and inferred influence scores, an inferred score for how well differences in two cell states can be explained by a TF, in primary MLL-AF9-driven samples when compared to MLL-AF9-driven THP-1 cells. The color and size of the individual dots represent an approximation for the number of target genes that are calculated from the number of edges in differential GRNs. The dotted line represents a visual cut-off for highlighting the top ranked TFs. (**b**) Identification of TFs in MLL-AF9-driven THP-1 cells compared to primary MLL-AF9-driven samples, visualized similarly to (a). (**c**) Identification of TFs in MLL-AF9 AML compared to primary CD14 + monocytes, visualized similarly to (a).

Although cell lines provide excellent models to study cell transformation, their genetic makeup and gene regulatory networks may differ from primary cells. Unfortunately, in most cases, availability of primary AML material is limited. To identify important differential TFs between cell line- or primary- associated MLL-AF9-driven gene programs, we performed ANANSE-CAGE again in two directions. First, TFs that drive primary MLL-AF9 immortalisation was determined, with THP-1 cells as source cell type. This revealed MECOM, MEIS1, and ERG (Fig. 5a and Supplementary Table S1), that have been reported to contribute to transformation and aggressiveness of MLL-AF9-driven leukemia^45^. Furthermore, we predicted MLL-AF9 targets FOS, FOSB^46^, and KLF6. The latter of which has not yet been described in MLL-AF9 AML. Second, we performed ANANSE-CAGE to predict TFs unique to THP-1 by comparing GRNs between primary samples (source type) and THP-1 cells (target type). Surprisingly, most of the top ranked factors were associated with neural and craniofacial development (BARX1, BHLHE22, OTX1, SP9, ALX1, and HMX3) (Fig. 5b and Supplementary Table S1)^47,48^. These results give us more insights into which TFs regulate key gene programs to keep THP-1 cells indefinite in cell culture, in contrast to primary AML cells.

To extend the approach on primary material, we compared our primary MLL-AF9 AML samples with publicly available primary CD14 + monocytes, and vice versa (Fig. 5c, Supplementary Fig. S3f,g,h,i and Supplementary Table S1). We analysed which TFs are predicted to be enriched in MLL-AF9 blasts and revealed a set of TFs that are well documented to have a role in MLL-AF9 leukemias, such as MECOM (EVI1), but also HOXA9, and HOXA7^6,49^. Interestingly, this also identified a highly conserved member of the MYB family MYBL2, reported to be associated with poor patient outcome in various cancers, including AML^50^. In summary, we predicted TFs differentially important between cell line- or primary- associated MLL-AF9-driven gene programs, and in primary MLL-AF9 AML. Indeed, many of these predicted TFs have been well described in literature in the context of MLL-AF9 AML. In addition, ANANSE-CAGE predicted novel key TFs providing an instructive set of factors that can be further explored in the future.

## Discussion

Here, to allow advanced predictability while retaining minimal experimental requirements, we developed a computational framework that predicts important TFs in hematopoietic development and disease with a bidirectional transcription network-based prediction algorithm using only CAGE-seq data as input.

We report correlation of enhancer-specific histone modifications with CAGE-defined bidirectional transcription initiation. Indeed, by predicting TFs key in keratinocyte conversion we were able to predict TP63 as the highest influential TF, which predominantly binds to enhancers^51^. Enhancer associated genomic regions are not only important in regulating tissue-specific gene expression but also in disease^52^.

We applied our framework to cell type conversion strategies, differentiation, and disease. Though this framework can be applied to a widespread of organisms and cell systems, we highlighted its use in hematopoietic systems, e.g. erythroid differentiation, early myelopoiesis, THP1 immortalisation, and focus on key differences between cell line- or primary- associated MLL-AF9-driven TF expression. In particular, we revealed TFs key in MLL-AF9 AML, e.g. MECOM, HOXA9, and HOXA7^6,49^. High expression of MECOM is associated with high-risk AML and holds an important prognostic value. Currently, standardized treatment protocols are limited in inducing remission, especially for malignancies with high heterogeneity in genetic and cytogenetic abnormalities. Targeted therapy can therefore be an excellent alternative for patients that require a more untraditional treatment strategy. A computational framework as described here can serve a purpose in uncovering molecular mechanisms in disease and is therefore a great tool for generating novel entry-points in tackling a heterogeneous disease such as acute myeloid leukemia.

Importantly, our method does not require a large set of samples or multiple genome-wide measurements to provide high prediction performance. It does not depend on large collections of reference data, making it a profitable method to use with new data. The rich epigenomic and transcriptomic landscape that is important in understanding hematological disease can be examined using just a single transcriptome-based Next Generation Sequencing technique. The framework is widely applicable to other cell systems and organisms. ANANSE-CAGE is open-sourced and available to expand upon (https://github.com/vanheeringen-lab/ANANSE).

Naturally, there are limitations associated with our approach. First and foremost, the resourcefulness of this framework is dependent on the number of biological replicates and heterogeneity between these replicates. We apply a strict filtering strategy that removes lowly expressed CAGE tags and preserve CAGE tag clusters that are expressed in 2 or more samples. Ideally three biological replicates is advised to identify high confident bidirectional regions, though not necessary. Highly heterogeneous samples will reduce the amount of high confidence bidirectional regions and will therefore limit the enhancer-target interaction prediction. Notably, this method makes use of differential networks, so determining the source and target cell type is important. TFs can be inferred as unimportant when these TFs are similarly expressed in both cell types. Also, this network-based method determines enhancer-target interaction based on distance and not genomic distance screenings. Besides, only TFs with a role in activation are implemented in this framework. Lastly, the best performing model for logistic regression includes assemblies hg19 and hg38. Other assemblies will use a lower performing model until more assemblies are supported. Though novel predicted TFs need to be explored in more dedicated assays, this computational workflow is powerful in providing insights into TF mediated regulatory mechanisms.

In conclusion, we demonstrated a low-requirement and high performing computation framework to predict important TFs in hematopoietic development and disease. By exploiting bidirectional transcription initiation we can identify epigenomic as well as transcriptomic landscapes important for determining gene regulatory networks. We demonstrate the wide applicability and informativity of this technique in hematopoietic systems, showcasing an open-sourced and powerful tool for understanding molecular regulatory mechanisms and disease.

## Materials and methods

### Cell culture, RNA extraction, and RNA-seq

The leukemic cell line THP-1 (ATCC) was maintained in RPMI 1640 (Gibco, Thermo Fisher Scientific) supplemented with 10% heat-inactivated fetal calf serum (FCS) at a density between 2E5 and 1E6. The cells were kept in a humidified incubator at 37 °C in 5% CO_2_ and tested negative for mycoplasma. Cells were treated with 0.2 µM GSK-LSD1 (Signa-Aldrich) for 8 h and RNA was isolated using Mini RNA Isolation II Kit (Zymo Research).

Library generation was performed on 500 ng RNA (singlicate) using KAPA RNA HyperPrep Kit with RiboErase (HMR) (Kapa Biosystems) with RNA fragmentation of approximately 300 bp fragments for 6 min at 94 °C. Library size distribution was measured using High Sensitivity DNA analysis (Agilent) on an Agilent 2100 Bioanalyzer and its corresponding software. Libraries with average sizes with approximately 300–400 bp were used for sequencing with a NextSeq 500 system (Illumina).

The fastq files (GSE204710) were mapped to the reference human genome hg38 through the Seq2science pipeline (https://github.com/vanheeringen-lab/seq2science) (STAR as default aligner).

### CAGE-seq

CAGE libraries were prepared using 2 µg total RNA from wildtype and GSK-LSD1 treated THP-1 cells and from blast cells of three MLL-AF9 AML patients. Kabushiki Kaisha DNAFORM performed CAGE sequencing on an Illumina system and basic data pre-processing. Fastq files (GSE204708, GSE204707) were mapped using the BWA and HISAT2 aligners to hg38. Post-mapping processing involved converting BAM to CTSS BED files (https://fantom.gsc.riken.jp/5/sstar/Protocols:HeliScopeCAGE_read_alignment). Genome coordinates were converted between assemblies using UCSC’s utility program liftOver.

### Publicly available datasets

CAGE-seq data for GM12878, HeLaS3, HepG2, H1-hESC, K562, fibroblasts (skin), astrocytes (cerebral cortex), hepatocytes, human iPSCs, keratinocytes, macrophages, K562 Hemin induced time course, and primary CD14 + monocytes were obtained from the FANTOM5 collection (https://fantom.gsc.riken.jp/5/sstar/Browse_samples)^53^. Publicly available ChIP-seq data was downloaded from the ENCODE portal (https://www.encodeproject.org)^22^. All datasets (hg19) are summarized in Supplementary Table S3.

### Bidirectional regions characterisation

Bidirectional regions were characterized using deepTools^54^, intervene^55^, and GREAT^56^. Absolute distance to TSSs were determined by associating genomic regions with single nearest gene. Also, genomic regions not associated with any genes were removed. CAGE tags were visualised using the UCSC Genome Browser^57^.

### CAGE-seq analyses

CAGE tags were analysed with the R package CAGEfightR v1.14.0^16^. CTSSs were normalized (TPM), pooled, and pre-filtered with default settings. Subsequently, tag clusters were quantified using a slice-reduce method. Tag Clusters (TCs) expressed in at least 2 or more libraries were kept and noise or weakly expressing TCs were discarded (> 1 TPM). CTSSs within a range of 20 bp on the same strand were systematically clustered into unidirectional clusters. Bidirectional transcription was established using a balance threshold of 0.95 and a window size of 201 bp. Unidirectional TCs were summed per gene annotation (TxDb objects) and used for differential expression analysis (DESeq2)^58^. For more details, a R markdown script is freely available at https://github.com/vanheeringen-lab/ANANSE-CAGE.

### ANANSE model training

The model training for bidirectional regions is based on the model training of ANANSE for H3K27ac and ATAC-seq data, described previously by Xu et al.^15^. To train the models we used bidirectional regions for 230 TFs from ReMap 2022 in five cell lines from FANTOM5: GM12878, HeLaS3, HepG2, H1-hESC, and K562^21^. Only factors defined in Lovering et al. were used^59^. The window size was normalized to 200 bp, centred on the middle of each bidirectional region. Bidirectional TPMs were log-transformed and quantile normalized. To evaluate the model training, including the general model, we performed cross-validation by held-out chromosomes and cell types, respectively. PR AUC scores were calculated using only enhancers located on held-out chromosomes in held-out cell types. For unavailable TF data, we trained a general model so that we can implement the model training for all TFs.

A standard logistic regression model, as implemented in scikit-learn^60^, was used to predict TF binding using three types of data: enhancer intensity score in TPM (bidirectional regions), TF motif scores, and the average ReMap 2022 ChIP-seq coverage^21^. Equation (1) was used to calculate the binding score.

1 $$ \log \frac{{p_{f,l} }}{{1 - p_{f,l} }} = \beta_{1} S_{f,l} + \beta_{2} E_{CAGE,l} + \beta_{3} E_{ChIP,l} $$

Here, ${p}_{f,l}$ is the probability of a transcription factor $f$ binding to enhancer $l$. ${S}_{f,l}$ is the highest motif z-score of all motifs associated with transcription factor $f$ in enhancer $l$, determined by using GimmeMotifs^61^. ${E}_{CAGE,l}$ is the enhancer intensity of enhancer $l$⁠, based on log-transformed and quantile normalized bidirectional CAGE-tags (TPMs). Lastly, ${E}_{ChIP,l}$ is the average ReMap 2022 ChIP-seq signal.

### ANANSE-CAGE implementation

Binding, interaction (including GRNs), and influence scores were calculated as previously described by Xu et al.^15^. TF prediction using CAGE-defined bidirectional regions were implemented in the binding prediction module of ANANSE. Putative enhancer regions were normalized to a window size of 200 bp, centred on the middle of each region and TPM values were log-transformed and scaled. In addition, ReMap 2022 ChIP-seq average coverage was automatically determined for assemblies hg19 and hg38. Gene regulatory networks were determined using summed unidirectional TCs per gene. Finally, influence scores were calculated using 500 k edges. ANANSE source code including the CAGE module is available from https://github.com/vanheeringen-lab/ANANSE. Jupyter notebooks for supporting analyses and a Rmarkdown file describing CTSS processing are provided at https://github.com/vanheeringen-lab/ANANSE-CAGE.

### Hemin induced differentiation time course analysis

We systematically calculated the influence scores for each timepoint (3.5 h, 24 h, 96 h) by comparing the respective timepoints to timepoint 0 h. K-means clustering was performed ComplexHeatmap^62^. Elbow method was used to determine the optimal value of k in k-means clustering (Supplementary Fig. S2). For this, R packages factoextra and nbClust were used^63,64^. The influence scores of predicted TFs per cluster were averaged to depict their temporal importance in hemin induced erythroid differentiation.

## Supplementary Information

Supplementary Information 1.

Supplementary Information 2.

Supplementary Information 3.

Supplementary Information 4.

## Data Availability

The datasets analyzed during the current study are available in the Gene Expression Omnibus (GEO) repository GEO Series GSE204711. ANANSE source code including the CAGE module is available from https://github.com/vanheeringen-lab/ANANSE. Jupyter notebooks for supporting analyses and a Rmarkdown file describing CTSS processing are provided at https://github.com/vanheeringen-lab/ANANSE-CAGE.

## References

[1] Mitsis T (2020). Transcription factors and evolution: An integral part of gene expression (Review). World Acad. Sci. J..

[2] Lambert SA (2018). The human transcription factors. Cell.

[3] Iwasaki H (2006). The order of expression of transcription factors directs hierarchical specification of hematopoietic lineages. Genes Dev..

[4] Göttgens B (2015). Regulatory network control of blood stem cells. Blood.

[5] Martens JHA, Stunnenberg HG (2010). The molecular signature of oncofusion proteins in acute myeloid leukemia. FEBS Lett..

[6] Birdwell C (2021). EVI1 dysregulation: Impact on biology and therapy of myeloid malignancies. Blood Cancer J..

[7] Storti F (2017). 529 European hematology association haematologica. Haematol..

[8] Gaidzik VI (2016). RUNX1 mutations in acute myeloid leukemia are associated with distinct clinico-pathologic and genetic features. Leukemia.

[9] Delgado FM, Gómez-Vela F (2019). Computational methods for gene regulatory networks reconstruction and analysis: A review. Artif. Intell. Med..

[10] Kim Y, Hao J, Gautam Y, Mersha TB, Kang M (2018). DiffGRN: Differential gene regulatory network analysis. Int. J. Data. Min. Bioinform..

[11] D’Alessio AC (2015). A systematic approach to identify candidate transcription factors that control cell identity. Stem Cell Rep..

[12] Yin W, Mendoza L, Monzon-Sandoval J, Urrutia AO, Gutierrez H (2021). Emergence of co-expression in gene regulatory networks. PLoS ONE.

[13] Marbach D (2012). Predictive regulatory models in Drosophila melanogaster by integrative inference of transcriptional networks. Genome Res..

[14] Rackham OJL (2016). A predictive computational framework for direct reprogramming between human cell types. Nat. Genet..

[15] Xu Q (2021). ANANSE: An enhancer network-based computational approach for predicting key transcription factors in cell fate determination. Nucleic Acids Res..

[16] Thodberg M, Thieffry A, Vitting-Seerup K, Andersson R, Sandelin A (2019). CAGEfightR: Analysis of 5′-end data using R/Bioconductor. BMC Bioinform..

[17] de Klerk E, AC’t Hoen P (2015). Alternative mRNA transcription, processing and translation: Insights from RNA sequencing. Trends Genet..

[18] Sartorelli V, Lauberth SM (2020). Enhancer RNAs are an important regulatory layer of the epigenome. Nat. Struct. Mol. Biol..

[19] Core LJ (2014). Analysis of nascent RNA identifies a unified architecture of initiation regions at mammalian promoters and enhancers. Nat Genet.

[20] Kang Y, Kim YW, Kang J, Kim AR (2021). Histone H3K4me1 and H3K27ac play roles in nucleosome eviction and eRNA transcription, respectively, at enhancers. FASEB J..

[21] Hammal F, de Langen P, Bergon A, Lopez F, Ballester B (2022). ReMap 2022: A database of human, mouse, Drosophila and Arabidopsis regulatory regions from an integrative analysis of DNA-binding sequencing experiments. Nucleic Acids Res..

[22] Sloan CA (2016). ENCODE data at the ENCODE portal. Nucleic Acids Res.

[23] Feng R (2008). PU1 and C/EBPα/β convert fibroblasts into macrophage-like cells. Proc. Natl. Acad. Sci. U. S. A..

[24] Kurita M (2018). In vivo reprogramming of wound-resident cells generates skin epithelial tissue. Nature.

[25] Caiazzo M (2015). Direct conversion of fibroblasts into functional astrocytes by defined transcription factors. Stem Cell Rep..

[26] Yu J (2007). Induced pluripotent stem cell lines derived from human somatic cells. Science.

[27] Takahashi K (2007). Induction of pluripotent stem cells from adult human fibroblasts by defined factors. Cell.

[28] Nakamori D, Akamine H, Takayama K, Sakurai F, Mizuguchi H (2017). Direct conversion of human fibroblasts into hepatocyte-like cells by ATF5, PROX1, FOXA2, FOXA3 and HNF4A transduction. Sci. Rep..

[29] Simeonov KP, Uppal H (2014). Direct Reprogramming of Human Fibroblasts to Hepatocyte-Like Cells by Synthetic Modified mRNAs. PLoS ONE.

[30] Zhenjiawang Z (2018). BART: A transcription factor prediction tool with query gene sets or epigenomic profiles. Bioinformatics.

[31] Qin Q (2020). Lisa: inferring transcriptional regulators through integrative modeling of public chromatin accessibility and ChIP-seq data. Genome Biol.

[32] Alvarez MJ (2016). Functional characterization of somatic mutations in cancer using network-based inference of protein activity. Nature Genet..

[33] Cahan P (2014). Cell net: Network biology applied to stem cell engineering. Cell.

[34] Tallack MR (2010). A global role for KLF1 in erythropoiesis revealed by ChIP-seq in primary erythroid cells. Genome Res..

[35] Garçon L (2005). Gfi-1B plays a critical role in terminal differentiation of normal and transformed erythroid progenitor cells. Blood.

[36] Wontakal SN (2012). A core erythroid transcriptional network is repressed by a master regulator of myelo-lymphoid differentiation. Proc. Natl. Acad. Sci. U. S. A..

[37] Vuckovic D (2020). The polygenic and monogenic basis of blood traits and diseases. Cell.

[38] Chen MH (2020). Trans-ethnic and ancestry-specific blood-cell genetics in 746,667 individuals from 5 global populations. Cell.

[39] Cusan M (2018). LSD1 inhibition exerts its antileukemic effect by recommissioning PU.1- and C/EBPα-dependent enhancers in AML. Blood.

[40] Fang J (2017). Upregulation of CD11b and CD86 through LSD1 inhibition promotes myeloid differentiation and suppresses cell proliferation in human monocytic leukemia cells. Oncotarget.

[41] Lynch JT, Cockerill MJ, Hitchin JR, Wiseman DH, Somervaille TCP (2013). CD86 expression as a surrogate cellular biomarker for pharmacological inhibition of the histone demethylase lysine-specific demethylase 1. Anal Biochem..

[42] Feinberg MW (2007). The Kruppel-like factor KLF4 is a critical regulator of monocyte differentiation. EMBO J..

[43] Ciau-Uitz A, Wang L, Patient R, Liu F (2013). ETS transcription factors in hematopoietic stem cell development. Blood Cells Mol. Dis..

[44] Bretones G, Delgado MD, León J (2015). Myc and cell cycle control. Biochim. Biophys. Acta.

[45] Stavropoulou V, Peters AHFM, Schwaller J (2018). Aggressive leukemia driven by MLL-AF9. Mol. Cell. Oncol..

[46] Fleischmann KK, Pagel P, Schmid I, Roscher AA (2014). RNAi-mediated silencing of MLL-AF9 reveals leukemia-associated downstream targets and processes. Mol Cancer.

[47] Larsen KB, Lutterodt MC, Møllgård K, Møller M (2010). Expression of the Homeobox Genes OTX2 and OTX1 in the Early Developing Human Brain. J. Histochem. Cytochem..

[48] Nagel S, Pommerenke C, Meyer C, MacLeod RAF, Drexler HG (2020). Aberrant expression of NKL homeobox genes HMX2 and HMX3 interferes with cell differentiation in acute myeloid leukemia. PLoS ONE.

[49] Ayton PM, Cleary ML (2003). Transformation of myeloid progenitors by MLL oncoproteins is dependent on Hoxa7 and Hoxa9. Genes Dev.

[50] Xin Z (2022). (2022) Elevated expression of the MYB proto-oncogene like 2 (MYBL2)-encoding gene as a prognostic and predictive biomarker in human cancers. Math. Biosci. Eng..

[51] Qu J (2018). Mutant p63 affects epidermal cell identity through rewiring the enhancer landscape. Cell Rep..

[52] Claringbould A, Zaugg JB (2021). Enhancers in disease: Molecular basis and emerging treatment strategies. Trends Mol. Med..

[53] Noguchi S (2017). FANTOM5 CAGE profiles of human and mouse samples. Sci. Data.

[54] Ramírez F (2016). deepTools2: A next generation web server for deep-sequencing data analysis. Nucleic Acids Res..

[55] Khan A, Mathelier A (2017). Intervene: A tool for intersection and visualization of multiple gene or genomic region sets. BMC Bioinform..

[56] McLean CY (2010). GREAT improves functional interpretation of cis-regulatory regions. Nat. Biotechnol..

[57] Kent WJ (2002). The human genome browser at UCSC. Genome Res.

[58] Love MI, Huber W, Anders S (2014). Moderated estimation of fold change and dispersion for RNA-seq data with DESeq2. Genome Biol..

[59] Lovering RC (2021). A GO catalogue of human DNA-binding transcription factors. Biochim. et Biophys. Acta Gene Regul. Mech..

[60] Pedregosa Fabianpedregosa F (2011). Scikit-learn: Machine learning in Python gaël varoquaux bertrand thirion vincent dubourg alexandre passos pedregosa, varoquaux, gramfort et al matthieu perrot. J. Mach. Learn. Res..

[61] van Heeringen SJ, Veenstra GJC (2011). GimmeMotifs: A de novo motif prediction pipeline for ChIP-sequencing experiments. Bioinformatics.

[62] Gu Z, Eils R, Schlesner M (2016). Complex heatmaps reveal patterns and correlations in multidimensional genomic data. Bioinformatics.

[63] Irnawati I, Riswanto FDO, Riyanto S, Martono S, Rohman A (2021). The use of software packages of R factoextra and FactoMineR and their application in principal component analysis for authentication of oils. Indones. J. Chemom. Pharm. Anal..

[64] Charrad M, Ghazzali N, Boiteau V, Niknafs A (2014). NbClust: An R package for determining the relevant number of clusters in a data set. J. Stat. Softw..

