# Supplementary material for: Identification of transcription factors dictating blood cell development using a bidirectional transcription network-based computational framework
Source: Sci Rep. 2022 Nov 4;12:18656. doi: 10.1038/s41598-022-21148-w (PMC9636203; doi:10.1038/s41598-022-21148-w)
Supplement: Supplementary file 1 — Supplementary Information 1. [file 41598_2022_21148_MOESM1_ESM.pdf]

Identification of transcription factors dictating blood cell development using a bidirectional transcription network-based computational framework

B.M.H. Heuts[1,+], S. Arza-Apalategi[2,+], S. Frölich[3], S. M. Bergevoet[2], S. N. van den Oever[1], S. J. van Heeringen[3], B. A. van der Reijden[2,*,+], J. H. A. Martens[1,*,+]

[1] Department of Molecular Biology, Faculty of Science, RIMLS, Radboud University, 6525 GA Nijmegen, The Netherlands.

[2] Department of Laboratory Medicine, Laboratory of Hematology, Radboud Institute for Molecular Life Sciences (RIMLS), Radboud University Medical Center, 6525 GA Nijmegen, The Netherlands.

[3] Department of Molecular Developmental Biology, Faculty of Science, RIMLS, Radboud University, 6525 GA Nijmegen, The Netherlands.

* Bert.vanderReijden@radboudumc.nl, J.Martens@science.ru.nl

+ these authors contributed equally to this work.

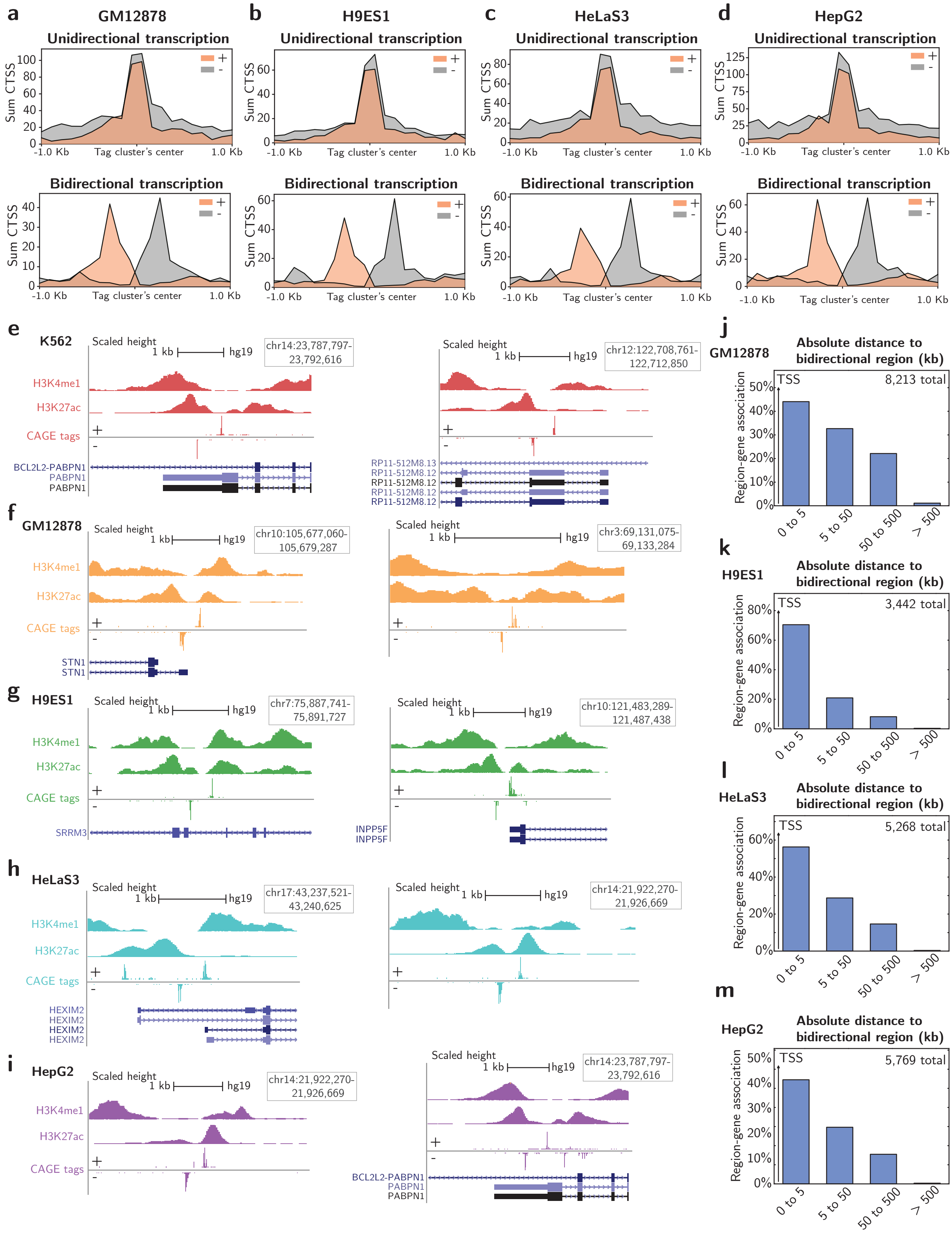

Supplementary Figure S1 - Characterizing CAGE-defined clusters in five cell lines.

(**a-d**) Profile plots of summed CAGE-defined Transcription Start Sites (CTSS) expression at unidirectional (top) and bidirectional (bottom) transcription clusters for GM12878 (a), H9ES1 (b), HeLaS3 (c), and HepG2 (d) samples. The orange color depicts the forward DNA strand (+) and in grey the reverse (-).

(**e-i**) UCSC Genome browser illustration of CAGE-seq tags signifying bidirectional transcription, H3K4me1 and H3K27ac ChIP-seq signals for K562 (e), GM12878 (f), H9ES1 (g), HeLaS3 (h), and HepG2 (i).

(**j-m**) Bar graphs representing absolute distance to bidirectional regions. Y-axis shows the fraction of regions associated with genes for GM12878 (j), H9ES1 (k), HeLaS3 (l), and HepG2 (m).

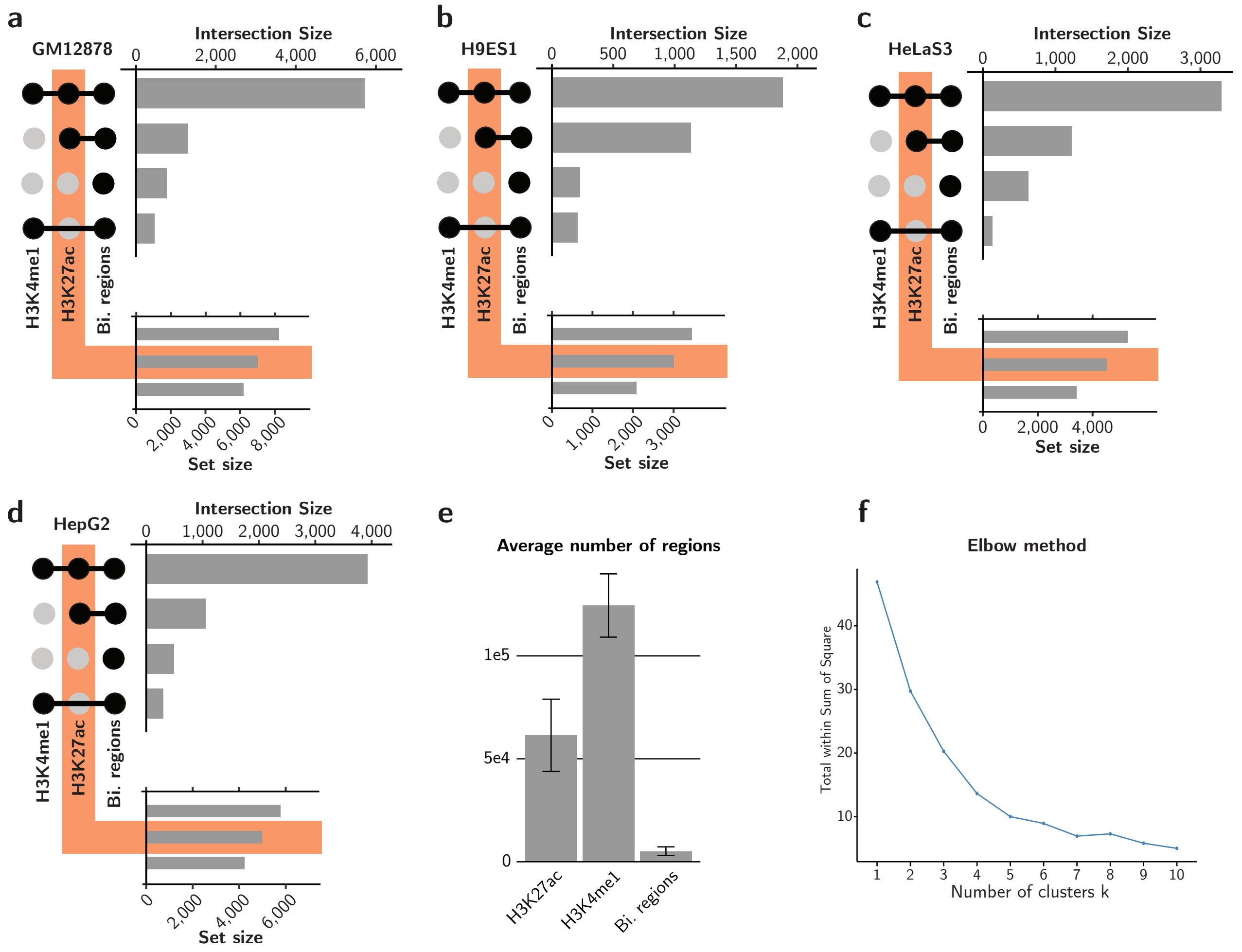

Supplementary Figure S2 - Determining intersecting bidirectional regions with enhancer-asso-ci-ated ChIP-seq regions, number of regions detected with CAGE- or histone ChIP-seq, and k-means optimization.

(**a-d**) Genome-wide co-occurrence (intersection size) of H3K4me1, H3K27ac, and bidirectional regions (Bi. regions) in GM12878 (a), H9ES1 (b), HeLaS3 (c), and HepG2 (d) samples. The black dots represent intersection between the two ChIP-seq marks and bidirectional transcription. The set size represent the total amount of regions included.

(**e**) Bar chart illustrating the average (n=5) number of regions detected for histone ChIP-seq (H3K27ac/H3K4me1) and CAGE-seq (bidirectional regions) in K562, GM12878, H9ES1, HeLaS3, and HepG2. Error bars represent standard deviation.

(**f**) Line graph depicting total within sum of square on the y-axis and the number of clusters k on the x-axis.

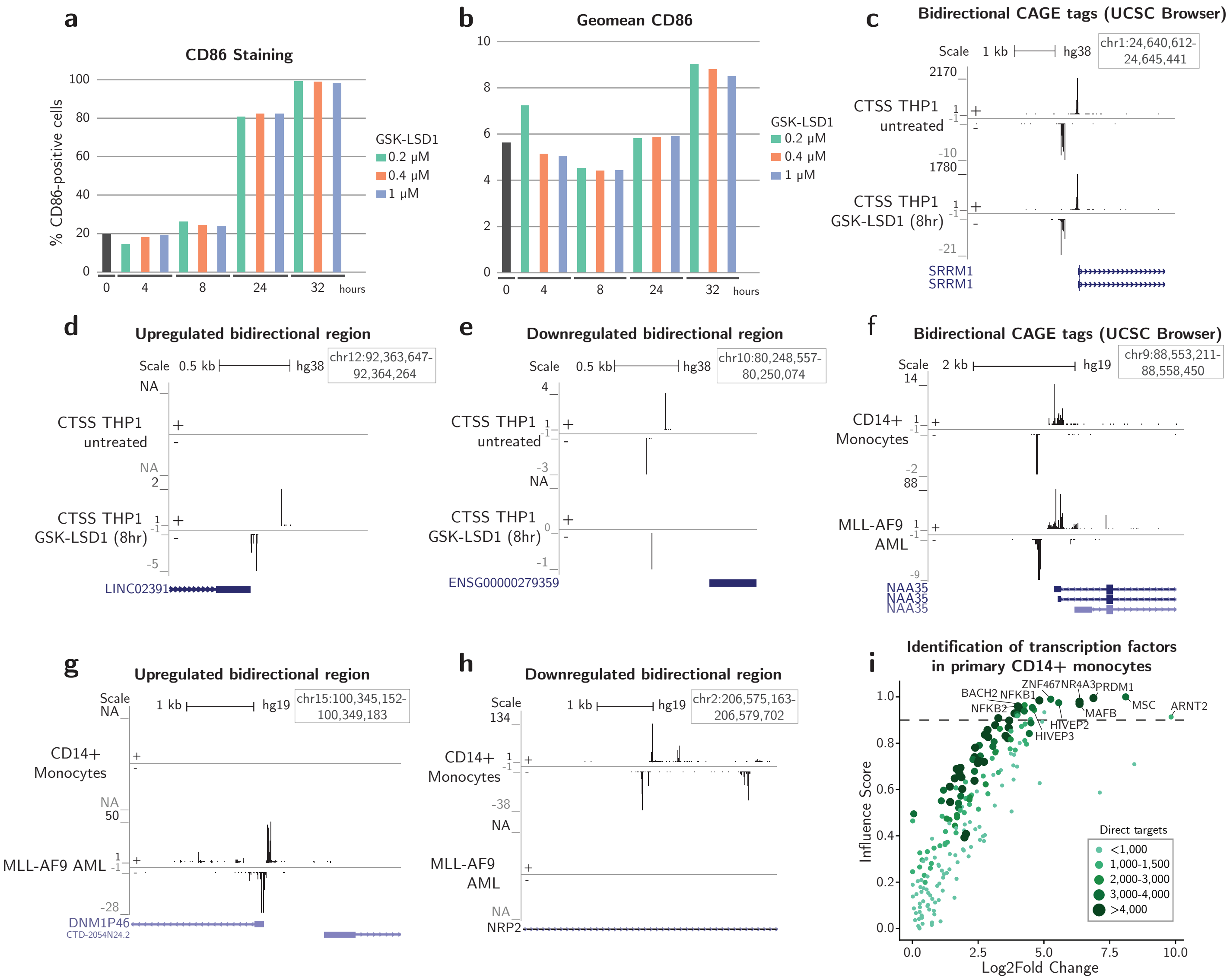

Supplementary Figure S3 - Determining CD86 expression, examples of (temporal) bidirectional regions, and key TFs in CD14+ monocytes.

(**a**) Bar graph illustrating percentage of THP-1 cells presenting cell-surface myeloid marker CD86 on five timepoints: 0, 4, 8, 24, 32 hours. Colors depict different GSK-LSD1 concentrations.

(**b**) Bar graph showing geomean of CD86 presenting THP-1 cells for five timepoints: 0, 4, 8, 24, 32 hours. Colors depict different GSK-LSD1 concentrations.

(**c-e**) UCSC Browser tracks of CAGE-seq tags representing bidirectional transcription in wildtype and GSK-LSD1 induced THP-1 cells. (c) depicts bidirectional transcription in both conditions, whereas (d) shows an example of an upregulated bidirectional region upon GSK-LSD1 induction and (e) shows downregulation of another exemplified bidirectional regions.

(**f-h**) UCSC Browser tracks of CAGE-seq tags representing bidirectional transcription in primary CD14+ monocytes and primary MLL-AF9 AML. (c) depicts bidirectional transcription in both conditions, whereas (d) shows an example of an upregulated bidirectional region upon GSK-LSD1 induction and (e) shows downregulation of another exemplified bidirectional regions.

(**h**) Scatterplot depicting log2 fold change and inferred influence scores, an inferred score for how well differences in two cell states can be explained by a TF, in primary CD14+ monocyte compared to MLL-AF9 AML. The color and size of the individual dots represent an approximation for the number of target genes that are calculated from the number of edges in differential GRNs. The dotted line represents a visual cut-off for highlighting the top ranked TFs.
